# Benefits of Digital Mental Health Care Interventions for Correctional Workers and Other Public Safety Personnel: A Narrative Review

**DOI:** 10.3389/fpsyt.2022.921527

**Published:** 2022-07-08

**Authors:** Elnaz Moghimi, Yuliya Knyahnytska, Mohsen Omrani, Niloofar Nikjoo, Callum Stephenson, Gina Layzell, Alexander Ian Frederic Simpson, Nazanin Alavi

**Affiliations:** ^1^Department of Psychiatry, Faculty of Health Sciences, Queen's University, Kingston, ON, Canada; ^2^Centre for Addiction and Mental Health, Toronto, ON, Canada; ^3^OPTT Inc., Toronto, ON, Canada; ^4^Centre for Neuroscience Studies, Faculty of Health Sciences, Queen's University, Kingston, ON, Canada

**Keywords:** behavioral therapy, correctional workers, depression, internet, mental health, online, psychotherapy, public safety personnel

## Abstract

Chronic exposure to stressors and potentially psychologically traumatic events contributes to the high prevalence of mental health disorders in correctional workers (CWs) and other public safety personnel (PSP). Digital mental health interventions are an accessible and scalable method of improving and maintaining the mental health of this population. The current review explores the benefits of digital mental health interventions for PSP–with a focus on CWs–and how these innovations can address the limitations in in-person mental health care. A systematic literature search of five databases (Medline, PsycInfo, Embase, CINAHL, Google Scholar) was conducted until March 2022. The search yielded 16 publications that focused on digital mental health interventions or care available to CWs and other PSP. The benefits of digital innovations were summarized into five categories which discussed (1) their ability to enhance accessibility and reduce stigma; (2) the provision of evidence-based and structured psychotherapy programs; (3) variability in the degree of therapist engagement; (4) the integration of proactive interventions; and (5) enhancing engagement by acknowledging unique experiences and interpersonal relationships. Although digital mental health technologies for CWs are still in their infancy, there is strong evidence to support their effectiveness in ameliorating symptoms of mental distress. Future research should consider how ethnicity, gender, culture, sexual orientation, and socioeconomic status can be integrated into these therapies and how the interplay between different stakeholders and organizations can impact the effectiveness of online therapies and programs.

## Introduction

Correctional workers (CWs) are public safety personnel (PSP) who work in prisons, jails, courthouses, and correctional centers and ensure the safety, security, and provision of services of staff and inmates (1). The complex and challenging work environment frequently exposes CWs to ongoing stressors and potentially psychologically traumatic events (PPTEs) (2–5). These factors increase the risk of occupational stress injuries (OSIs)–mental health disorders or conditions that result from PPTEs or stressful incidents in the workplace (6). Compared to the general public and other PSP sectors, there is a higher prevalence of mental health disorders in CWs (4, 7). A sample from Ontario, Canada (*n* = 1487), reported positive screens of 37% for major depressive disorder (MDD), 30.7% for post-traumatic stress disorder (PTSD), and 30.5% for generalized anxiety disorder (GAD) (8). Another study reported that 35.2% of CWs experience lifetime suicidal behavior with no significant difference based on years of service (9). Similar trends have also been observed in US-based CWs (10). In one report, since starting work in corrections, 49% of CWs experienced anxiety, 46% depression, 43% obesity or being overweight, 40% high blood pressure, 39% PTSD, and 23% alcohol use disorder (11).

The need for accessible mental health programs and therapies has substantially increased amid the COVID-19 pandemic (12). Prior to the pandemic, most mental health services offered by correctional institutions were delivered in person and focused on preventing or managing OSIs. For example, the Employee Assistance Program (EAP) is a frequently used voluntary service that aims to improve employee productivity by addressing personal and work-related concerns. EAPs can also help clients identify and resolve mental health-related concerns by providing assessments, short-term counseling, referrals, and follow-up services at no cost. Some institutions also provide critical incident stress management (CISM) and critical incident stress debriefing (CISD) programs for those at risk or exposed to PPTEs (13). Drawing on cognitive behavioral therapy (CBT) strategies, these programs promote treatment-seeking and support in affected individuals. Many external agencies also train PSP to provide peer mental health support. These programs promote emotional and social support, encouragement, and hope to colleagues exposed to workplace PPTEs (14). Training programs include CISM and CISD, peer support, psychological first aid, and trauma risk management (15). Lastly, the *Road to Mental Readiness Program* (R2MR) is a 160-min training program that assists PSPs in retaining their psychological well-being while working in high-risk occupational settings (16). The program uses CBT principles and psychoeducation to help individuals manage physiological stress responses and engage in psychological self-monitoring and attentional control (17).

Currently, there is limited evidence of the benefits of these interventions in mitigating PPTEs (15). In a review of 14 different mental health programs available to Canadian PSP, there was considerable variability in perceived intended use and delivery (13). Moreover, there was little evidence that the programs robustly impact symptoms of (OSIs), either positively or negatively (13). However, PSP training programs were associated with a greater willingness to access support and decreased odds of screening positive for most mental disorders (8). In another review and meta-analysis of well-being interventions for correctional officers (18), only nine studies met the exclusion criteria, and none were randomized controlled trials. The programs consisted of crisis interventions, psychoeducational programs, and an exercise program. Results indicated that the treatments did not affect stress and psychopathology. However, the authors noted that the interventions lacked a strong theoretical and context-specific basis, emphasizing a need for validated interventions based on sound models of psychological processes associated with well-being. The absence of well-established and targeted programs may partly explain the low treatment-seeking behaviors of CWs.

With the persistence of the pandemic, mental healthcare delivery has rapidly pivoted from in-person to online formats that adhere to physical distancing laws (19, 20). Digital health technologies enable mental health care to be primarily delivered through telephone, internet, or mobile applications. The online delivery of mental health programs and therapy is an easily scalable, affordable, and relatively accessible option (21). Some digital programs also demonstrate efficacy that is comparable to in-person care (22–25), even within PSP (26). Digital interventions can overcome many mental healthcare barriers as they are cost-effective, require less therapist time, and can be accessed from any private location at any time (19). These factors contribute to the appeal of online therapy and programs for CWs.

Exploring the mental health needs of CWs and their response to current in-person treatments can inform the development of internet therapies and programs tailored specifically for this population. The objective of the current narrative review was to explore the benefits of digital interventions for PSP and how they can address barriers that frequently exist in in-person care. Although a sizable proportion of empirical research does not distinguish the PSP sectors, the application of the findings to CWs specifically are discussed. The findings have been summarized into five broad categories. The benefits describe how digital mental health delivery can adequately address barriers and improve the quality of mental health care for this population.

## Methods

The present review adhered to PRISMA-P (Preferred Reporting Items for Systematic Reviews and Meta-analyses) guidelines (27, 28). A systematic literature search was conducted on the web databases Medline (OVID), PsycInfo (EBSCO), Embase (OVID), CINAHL (EBSO), and Google Scholar. Combinations of MeSH (Medical Subject Headings) and keywords related to correctional workers, digital interventions, and mental health were used in syntax with other Boolean terms to develop the search algorithms. A full list of terms can be found in Supplementary Table 1.

Peer-reviewed studies published in English and until March 2022 were included. Studies were included if they focused on digital mental health interventions or training available to correctional employees within all sectors. Studies pertaining to prisoner mental health or trauma were excluded. Studies on PSP that did not recruit or report sampling CWs were also included if they defined CWs as being part of PSP and/or reviewed programs that have been used by CWs in other studies.

## Results

After removing duplicates, 2, 468 studies were screened and 16 studies were identified from the search (Supplementary Figure 1). The majority of the studies were reviews (n = 5), followed by mixed-methods (*n* = 4) and qualitative (*n* = 3) studies (Supplementary Figure 2). Most of the research studies sampled from or examined an intervention in Canadian PSP (n = 10). One of these studies (29) was conducted using members of the Canadian Armed Forces. This study was included in the review since the program they assessed is available to CWs and other PSP (7). One study (30) recruited PSP but the sample did not include CWs and two studies did not specify the PSP sectors recruited (26, 31). These papers were also included for the same reason as the (29) study. Studies exclusively reviewing digital interventions only focused on mental health apps for PSP (*n* = 3) (32–34). These studies were included since they acknowledged CWs as being part of PSP. Lastly, all papers were published between 2019 and 2022, with the majority published in 2021 (*n* = 9). A full summary of the papers can be found in Supplementary Table 2.

### Summary of Benefits

#### Enhanced Accessibility and Reduced Stigma

Shift work and long hours can compound stress and make it challenging for CWs to find the time to seek care (35, 36). In a study of PSP perceptions of electronic CBT (e-CBT), time flexibility and convenience were the most commonly cited benefits, followed by anonymity and privacy (37). Qualitative data indicate that although CWs prefer to work with a therapist, they favor off-site assessments to safeguard privacy (35). Specifically, the fear of breached confidentiality and others becoming aware of their mental health status has been reported as a barrier to treatment-seeking in CWs (35). This phenomenon is also reflected in the type of therapy sought out by PSP. In one study, 74% of PSP would first access a spouse for mental health support (8). Many (43-60%) refrain from seeking professional support or only do so as a last resort (8). Online interventions address confidentiality concerns by eliminating the need for CWs to drive to a physical location, accessing therapy from wherever they are comfortable while better-preserving anonymity within their community and family.

The central importance of CWs accessing mental health care and sharing their concerns in a private space may be due to the high levels of workplace stigma surrounding mental health (7, 38). Although one study reported that CWs experience less stigma than other PSP sectors, it is still a significant problem in this population (7). Stigma can be broken down into two dimensions, public and self-stigma. Public stigma refers to societal prejudices and discrimination toward individuals seeking or receiving mental health care (39). Self-stigma occurs when individuals hold negative or self-condemning personal views (39–41). The correctional work culture frequently discourages visible emotional responses to trauma or stressors, reinforcing both types of stigmas (42). Most notably, the detection of mental health concerns can raise questions of whether the individual can perform their job duties. As a result, many CWs avoid seeking care for fear of shame, being discriminated against, labeled as pariahs, lazy, or weak, being discredited, or experiencing workplace repercussions (43, 44). Negative media portrayals and public perceptions of CWs as incompetent, brutal, and indifferent to human suffering can further fuel this stigma (45, 46). In CWs, a negative public image is a strong predictor of job stress and low community support (47). The outcome is that CWs avoid seeking timely mental health care, sharing their mental health concerns with others, and giving and receiving mental health support from their colleagues (38). Resultantly, stigma can bolster a work culture that normalizes toxic masculinity (i.e., socially regressive male traits), distress, isolation, and lack of support from colleagues–all of which can contribute to and worsen adverse mental health outcomes (42, 48).

One way that online interventions can reduce stigma is by increasing community and workplace mental health knowledge and recognition (35, 49, 50). Similar to other mass communication channels, internet-based mental health interventions have broad reach and can be accessed by large numbers of people (51, 52). In CWs, greater mental health knowledge is associated with reduced stigma and greater recognition of mental health needs and intent to use mental health services (7, 35). In a recent study comparing male and female correctional employees, male participants (38.6% of the sample) were significantly more likely to exhibit stigma toward individuals with mental disorders and were less likely to seek care if they developed a mental health disorder (49). Conversely, females were less likely to exhibit stigmatizing attitudes and more willing to seek mental health care–characteristics ascribed to their greater mental health knowledge and awareness of the stigma associated with mental health injuries. Although gender-based differences toward stigma exist, the study highlights mental health knowledge as critical to reducing stigma. Online interventions may counteract stigma by contributing to a more informed individual. Greater awareness in a less stigmatizing environment can encourage PSP to discuss mental health concerns, identify, self-report, and seek help, and take advantage of available services that promote positive mental health (7).

While the most salient features of online interventions are their convenience and accessibility, it is necessary to determine the type of online intervention appropriate for this population. For example, in some cases, the use of discreet online mental health interventions may perpetuate social avoidance. Active users of an online support group experienced less self-stigma and a greater likelihood of seeking formal support (53). On average, participants spent 1 h per visit, with 60% visiting more than once a day. However, increased frequency of use was associated with reduced self-stigma recovery and lower offline treatment-seeking. This pattern indicates that an overdependence on online support groups could be a form of social avoidance rather than a means of reducing stigma. Although there is only a modicum of research in CWs and other PSP (26), the highly stigmatizing mental health views in this profession may similarly reduce the benefits of certain online interventions with increased use. Therefore, researchers must ensure that programs and therapies encourage symptom improvements and reduce stigma rather than become an escape from reality (54–56). For this reason, the dissemination of online interventions based on empirical research is critical in this population.

#### Provision of Evidence-Based and Structured e-CBT Programs

Psychological interventions guided by relevant data produce cost-effective and efficacious psychiatric treatments (57). Implementing programs without rigorous evaluation may allocate already-limited funds toward ineffective or even harmful programs (58). Despite the demonstrated benefits of evidence-based in-person and online psychological interventions for CWs, the number of programs available are limited (18). Even so, the data indicates that the success of CISM and CISD programs can be attributed to their use of CBT techniques–an evidence-based treatment modality (8). CBT is the first-line treatment for several mental disorders, including anxiety, depression, and PTSD (59–61). Most available evidence-based programs for PSP and CWs utilize CBT since it can be easily adapted for different mental disorders and populations (25, 62). The therapy is typically divided into structured sessions consisting of psychoeducation, thought records, and behavioral experiments (63). At the end of each session, patients will complete and submit homework essay questions to reinforce and practice what they have learned. The homework is then sent to their therapist who reviews and provides personalized feedback on the patient's progress. e-CBT uses the same traditional concepts and skills as its in-person counterpart and demonstrates comparable efficacy (64). However, e-CBT is considered a more convenient form of care as it typically consists of weekly lessons and virtual therapist support (19, 64). In a recent study, PSP perceived e-CBT positively and believed it to be an appropriate means of addressing the community's high prevalence of mental health concerns (65). PSP also indicated that therapy should be tailored to address a range of symptoms while also acknowledging their focal concerns (65). In another study from the same research team, prospective clients (*n* = 259 PSP; 55 CWs) had a positive outlook on e-CBT and predicted a 55% improvement in their symptoms (66). In a study of 132 PSP, 93% reported that they would use e-CBT if they experienced mental health concerns (37).

In 2019, the Government of Canada initiated a National Action Plan (67) in which e-CBT was identified as a potential solution to overcome barriers to care and provide mental healthcare to PSP (68). To date, only two e-CBT programs have been tailored for CWs (69–71). One uses a modified version of the Well-being Course (PSP Well-being Course) for all PSP (70). The PSP Well-being Course consists of five psychoeducational lessons released over 8 weeks (72). These lessons focus on (1) the cognitive behavioral model and symptom identification; (2) thought monitoring and challenging; (3) de-arousal strategies and pleasant activity scheduling; (4) graded exposure; and (5) relapse prevention. The second study uses the Online Psychotherapy Tool (OPTT) to deliver unique programs for CWs at risk or diagnosed with PTSD, GAD, or MDD (69). OPTT is a virtual platform that offers online psychotherapy programs specific to different disorders and populations. Through weekly sessions, OPTT's 12-week program teaches core CBT concepts and skills. Both programs include case studies unique to the population of interest and homework that follows each session. In addition, both programs have varying degrees of therapist support. The use of evidence-based interventions strongly suggests greater improvements in the mental health of CWs–likely more than what is observed in current programs. Already, both programs have demonstrated beneficial effects in other populations (71–76). Initial outcome data (*n* = 83; 9 correctional workers) indicates that the PSP Well-being Course effectively treats symptoms of depression, anxiety, PTSD, panic disorder, and is moderately effective for treating anger (70). Furthermore, 54/62 (86%) of study participants found that the course made them feel more confident in their symptom-management abilities, and 61/62 (98%) of participants found that the course was worth their time. Qualitative data from a PSP sample with 11% CWs demonstrated positive client views of the PSP Well-being course and that the program was suitable for developing coping skills and normalizing mental health experiences (68).

Despite the positive views, virtual therapy is not the primary treatment choice for CWs (8). The relative novelty of e-CBT in this population may partly explain this preference. Since treatment outcome expectations are associated with treatment outcomes (77), participants may not be aware of the online program's benefits, thereby reducing its efficacy and use. Indeed, in a study exploring PSP perceptions of e-CBT, many of the questions posed by the participants indicated the need for educational material explaining the logistics of e-CBT and its delivery methods (37). Providing this information can also address low technological competence and familiarity issues that can contribute to attrition (31, 32, 34, 78–80) and may increase openness toward e-CBT amongst CWs.

#### Variable Therapist-Engagement

Most CWs prefer some degree of therapist or human support when partaking in mental health interventions (81). After psychologists, e-CBT with therapist assistance was ranked second to the most preferred treatment type in this population (81). Therapeutic alliance over the digital realm can mimic in-person therapies amongst PSP (82–85). At the same time, the degree of therapist interaction can be easily modified to meet the needs of the individual (76, 86). This factor makes online interventions a more cost-effective way to connect with therapists since they require less time commitment per client (87, 88). In a study of an e-CBT program for PSPs, most preferred therapist support once a week (74/83, 89%), followed by twice per week (6/83, 7.2%), and lastly on an as-needed basis (3/83, 4%) (70). Although a small portion of participants opted for optional online therapist support, in individuals with anxiety and depression, it is associated with lower completion rates and lower correspondence than those with standard weekly support (72). These findings indicate that online programs should offer some degree of therapist contact.

The therapeutic alliance is more likely to occur with synchronous video or telehealth compared to other delivery methods, since it provides a more direct opportunity for CWs to build relationships and trust with their therapists (89). The PSP Well-being course and OPTT utilize asynchronous text-based communication with the therapist. The benefit of these delivery formats is that there is some degree of therapist contact while still addressing many facets that make digital interventions appealing to CWs. For example, some PSP report difficulties finding a private space in their home (90), providing sensitive information online, or conveying and reading emotions and non-verbal cues (91, 92). Both e-CBT programs acknowledge these limitations by providing asynchronous treatments that are textual and can be completed over a few days. In addition, previous research has demonstrated that therapeutic alliance can be formed in e-CBT (93). However, to better address the impersonality that may arise, programs may benefit by integrating live and/or video-recorded options into their interventions. One study proposed the inclusion of initial in-person meetings with therapists to increase rapport and adherence (94).

Although confidentiality is a frequent concern of CWs, most prefer therapist-guided e-CBT to self-guided e-CBT (37, 68). The inclusion of a therapist in online mental health interventions can help mitigate feelings of isolation and enhance accountability (95). In addition, PSP report barriers to face-to-face treatment such as unaffordability, not being understood by the therapist or counselor, time constraints and concerns about mental health stigma (66). Moreover, the therapeutic alliance is strengthened when PSP work with therapists who have sufficient knowledge and experience working with this population (65, 68). A more customized and option-friendly approach is needed for both the intervention and the degree of therapist contact.

#### Integration of Proactive Interventions

Most studies on CW and PSP mental health interventions report favorable results (96–99) but vary in their strength of evidence (15). These studies frequently sample PSP with different employment lengths, mental health statuses, and sectors. The heterogeneity of results indicates that a one-size-fits-all approach may not be appropriate for this population.

Although evidence-based therapies like e-CBT may reduce burnout and stress (100, 101), they do not address the spectrum of mental health statuses that exists. It may be difficult for the general CW population to relate to the concepts discussed in reactive interventions like e-CBT. Conversely, proactive interventions are unlikely to be effective in individuals already struggling with clinical levels of psychological stress (15). Reactive interventions consisting of targeted digital mental health treatments can benefit employees at risk or diagnosed with mental health disorders. In contrast, proactive internet programs that promote CW well-being can help prevent mental health injuries, and assist with job retention (102, 103). Notably, proactive programs can equip individuals with skills and strategies they can use before or during stressful or traumatic events.

Some of the recommendations made by CWs (*n* = 67) for a healthier workforce included more training opportunities and programs, scheduled appointments with mental health professionals who can track their mental health status, and team-building opportunities that acknowledge interpersonal conflicts at work (35). These suggestions indicate that CWs embrace proactive interventions and see their value in the workplace. Proactive interventions can include promotion and education surrounding trauma and mental health to increase awareness, peer support and trauma-informed advocacy programs, access to mental health professionals, and increasing employee insurance benefits for mental health services (103). Many of these suggestions can be integrated into digital interventions.

Current mental health services available to CWs–including those in an online format- tend to be reactive and focus on staff who experience significant psychological distress. Programs for CWs struggling with daily stressors and challenges due to their adverse work environment are limited (16, 104). As a result, empirical research is lacking in CWs who do not meet clinical levels of cognitive deficit or mental health issues but still fall on the spectrum of psychological distress. A greater focus on proactive programming may benefit CW well-being (16). Moreover, proactive programs have been shown to enhance resilience and well-being and reduce emotional exhaustion, mental concerns, and burnout in CWs (105, 106). Many PSPs report a motivation to learn skills to manage their mental health symptoms, indicating the appropriateness of skills-based and resilience-building proactive treatments (66). Mental health training offered to US-based correctional officers varies considerably and ranges from 1.5 to 80 h. These training programs center around the safety and security of inmates and other officers and mainly focus on crisis intervention (84.62%) and general psychoeducation (46.15%) (107). However, standardized training programs specific to mental health and mental illness are lacking, as is research assessing their effectiveness in CWs (107). Similar trends have been observed in Canadian samples (8, 13). Cost-effective online interventions can offer users a wide selection of customizable proactive programs and help enhance the efficacy of current in-person programs without exhausting already-limited healthcare resources (108–110).

For example, leveraging digital technologies to complement the R2MR program is a suitable example of modifying a traditionally classroom-based proactive program to increase program length and enhance efficacy in a specific population. The low efficacy of the R2MR program in PSP (111, 112) indicates that a 160-min training program may be a great start to initiate mental health dialogue but may be insufficient in addressing the multifaceted mental health challenges in PSP sectors. Since the positive improvements and adaptive coping skills gained from these programs can diminish over time, continuous access may sustain beneficial effects (113). Repeatedly applying and practicing skills have demonstrated success in program retention and effectiveness (114). In line with this, the R2MR app complemented the current program by providing on-the-go training to help with stress management, short- and long-term performance and mental health outcomes, and encouraging treatment-seeking behaviors (17). The online program provided customizable life skills and access to additional care resources (29). Moreover, users could track their progress over time, receive reminders to practice resilience and executive functioning skills taught in the app, view multimedia and graphics to enhance engagement, and have immediate access to mental health information whenever they need it (29). Although the app is relatively novel, usability studies in members of the Canadian Armed Forces (CAF) have indicated that compared to civilian participants, CAF participants were more accepting of the app as a prescribed training tool and expressed a desire to view their progress relative to others (29, 33). Taken together, online interventions allow for the rapid implementation of a resource pool of proactive and reactive interventions that are cost-effective, easily customizable, and meet the diverse mental health needs of CWs (33).

#### Enhancing Engagement by Acknowledging Unique Experiences and Interpersonal Relationships

Despite the benefits of online interventions, low engagement, high dropout, and unsustained use are frequent problems (115–117). Online mental health interventions for PSP also have low participant adherence and completion, despite significantly reducing post-traumatic stress injuries and improving well-being, coping, and resilience (102). Although there are different facilitators of user engagement (118, 119), the two most relevant to CWs are (1) the importance of developing programs that acknowledge their unique experiences and (2) consideration of interpersonal relationships.

Given that each PSP sector experiences different types of traumas, stressors, and mental health symptomatology (4, 120), online interventions should be industry responsive and acknowledge the unique experiences of CWs. For example, the Before Operational Stress (BOS) program is a year-long CBT-based group program that aims to enhance positive mental health habits, self-awareness, and healthy relationships in early-career PSP (30). Qualitative findings demonstrate participants' positive views of the program. Additionally, small but statistically significant improvements were observed at 6 months in PTSD, anxiety, depression, and alcohol use symptoms, quality of life, stigma and perceived social support (30). The small effect may be due to the large individual variability present in the sample, highlighting the importance of developing occupationally responsive programs that meet the unique needs of CWs.

Although most studies amalgamate PSP (e.g., firefighters, police officers, correctional workers, paramedics), there are distinct differences in the job requirements and frequency and type of mental disorders and traumas experienced in each sector (4). For example, only CWs provide care, custody, and control of individuals housed in correctional facilities. Working in the same confined living space of prisoners substantially increases the risk of PPTEs and stress (121). Compared to other PSP, CWs display the highest rates of mental health disorders and suicidal behaviors despite having the greatest mental health knowledge, least stigma, and highest intentions to use mental health services (4, 7, 9). Generally, users prefer interventions that can be personalized to meet their unique preferences and needs, are accessible and interactive and offer support (122). Tailoring digital interventions to meet population-specific needs and interests can improve user engagement and instill a sense of ownership and control of health in users (123, 124). Based on these findings, interventions designed for all PSP are unlikely to have the same degree of efficacy and engagement as those that are sector-specific.

To enhance engagement, online interventions should draw on sector-specific examples and case studies that make it easier for users to relate their experiences. Because feelings of isolation and loneliness frequently co-occur with mental health concerns, tailored online interventions can improve engagement by reducing stigma and normalizing how common mental health concerns are amongst CWs and other PSPs (68). A recent e-CBT intervention for PSPs guided by Oinas-Kukkonen and Harjumaa's persuasive systems design (PSD) (125) demonstrated increased engagement in users (68). The PSD framework consists of 28 recommended design principles to enhance user engagement in online programs and interventions. Although the study did not indicate which principle resulted in the greatest engagement, it is speculated that the social learning principle may be the most important since it aligns closely with the social nature of the CW profession (43, 126). In addition to pursuing self-betterment and learning skills to independently manage their mental health, many PSP seek e-CBT to improve their family functioning and offer peer support to their colleagues–socially based motives (66). The importance of social learning is also evidenced by the relative success of peer support programs in PSP (15). At the same time, seeking social support may be problematic for some CWs as the associated stigma can jeopardize their social standing in the workplace (2). Therefore, CWs may be more inclined to engage with online programs that include real-world examples from their colleagues without the worry of sharing their private information as they would in a peer support setting.

## Discussion

The relatively recent rise in publications related to digital mental health technologies for CWs highlights the novelty of this research area. The majority of these studies were either reviews or explored the perceptions of PSP and CWs toward digital delivery of mental health care. Even so, preliminary evidence supports the success of these innovations in this population (70). Despite the scant research, the potential for online mental health interventions to mitigate the deleterious effects of occupational trauma and stress is well-demonstrated (66, 68, 103).

The high accessibility of online interventions can foster rapid dissemination of mental health knowledge–a critical factor to consider when tackling the problematic levels of stigma in the correctional profession. Due to the benefits of CBT (68–70), most of the interventions discussed in the current review focused on its online delivery. While reactive interventions like internet CBT can ameliorate the clinical symptoms of mental disorders, the findings indicate that CWs will also benefit from consistent access to proactive programs (102). Adapting traditionally in-person proactive and reactive programs into a digital format has shown some success in PSP (33), rendering it an area that demands greater investigation. Lastly, the findings indicate that engagement is one of the most fundamental factors to consider when developing online interventions for CWs, since most online programs are riddled with low adherence and completion (102). Although most of the current studies are not sector-specific, it is posited that CWs will better relate and engage with programs explicitly focused on their vocation. Additionally, virtual therapist contact will create a more personalized experience and will reduce the impersonality that is commonly cited as a limitation of online interventions.

An eclectic mix of digital mental health interventions with variable therapist involvement has the potential to foster precision mental health and improve care (127–129). Providing treatment options may bring forth a greater sense of control, autonomy and trust, making it more appealing for CWs who rely on these factors in the workplace (130, 131). In a sample of US CWs, 55% agree and 33% strongly agree that staff behavior influences the behavior of those incarcerated in the unit (11). Although more than half of the sample agreed that they rely on their coworkers to respond to an emergency, more than half also believed there was a lack of trust and teamwork in their work environment. While the stressful work environment is an inevitable part of this profession, providing accessible online resources that cater to variable mental health needs may contribute to a more positive work culture. In turn, a mentally healthy workforce may reduce the probability of biases and stress-related decision-making, contributing to a more compassionate work environment that is beneficial to both employees and offenders (132).

### Future Directions

The broad reach of digital technologies serves as the impetus for future interventions to not only acknowledge an individual's profession but perhaps other critical factors such as their ethnicity, gender, culture, sexual orientation, and socioeconomic status. In PSP exposed to PPTEs and other stressors, men are more likely to rely on families or spouses for social support and women are more likely to seek friend groups or reciprocity-based relationships and formal programs (133). While the relationship between gender and mental health has been somewhat outlined in CWs and other PSP, future research needs to consider how the other demographic factors impact the different facets of care. A systematic review included in this review highlighted the importance of examining gender, racial, and cultural factors when designing digital mental health interventions for PSP since they may result in differential outcomes, preferences, and needs (26, 134, 135).

The majority of the studies in this review focused on Canadian CWs and PSP–an emphasis that was a by-product of the locations in which the available studies were conducted. While it may be too early to determine the implications of digital mental health programs, their success may inspire other communities and populations to consider the mental health of those offering care and protection to prisoners. Future research should also consider how the interplay between different stakeholders and organizations can impact the effectiveness of online therapies and programs. For example, some skepticism has been detected in PSPs completing online courses that are government-sponsored (68). Future research is needed to determine how relationships with and the perceived credibility of the stakeholders and organizations offering online evidence-based programs can impact treatment outcomes. Researchers, institutions, and correctional facilities that develop and promote these online interventions may see greater benefits in users if they establish trust and conducive dialogue (136, 137).

Closer relationships and systematic trust within the community and PSP organizations can also generate a platform where users can provide constructive feedback on improving these evidence-based programs (137). For example, patients enrolled in an e-CBT program for depression and anxiety suggested improvements in the breadth of patient stories, course timeline, and matching therapist availability to patient needs (138). Perceived organizational support can moderate the deleterious effects of correctional work (139). To strengthen mutual trust and integrity, open conversation and acknowledging the patient voice are critical (35, 140). Hence, treatment, mental health, and well-being insights shared by CWs can not only offer invaluable feedback on improving online therapy but can subsequently strengthen trust, positive treatment outcomes, and promote a supportive and psychologically healthy (141, 142).

## Conclusion

Taken together, online interventions are a burgeoning method of obtaining mental health and well-being for CWs and other PSP- professions marked by high levels of occupational stress and trauma. The benefits explored in this review are necessary to inform the development of digital programs and therapies for this population. The high prevalence rates of mental disorders in CWs indicate that the current interventions and work environment require some degree of reform. It is noted that online interventions alone are not sufficient in initiating sustained change. To promote a mental health-positive work culture in corrections, all levels of the organizations–from government to administration to the individual–should be considered. Digital programs and therapies have the potential to assist with the multi-level shift in organizational mental health views. Ultimately, and as demonstrated in this review, what sets digital interventions apart from other delivery methods is that it provides a more personalized form of mental healthcare delivery that can actively adapt to an individual's clinical needs, goals, and lifestyles (143).

## Author Contributions

EM and NA were responsible for study design, conducting the systematic literature search, and writing the review. Subsequent drafts were edited and finalized by YK, MO, NN, CS, GL, and AIFS. All authors contributed to the article and approved the submitted version.

## Funding

This study was funded by the Canadian Institutes of Health Research Operating Grant (File #: RN410776-433679). The funding agency had no role in the writing of this review.

## Conflict of Interest

NA and MO cofounded an online care delivery platform (i.e., OPTT) and have ownership stakes in OPTT Inc. The remaining authors declare that the research was conducted in the absence of any commercial or financial relationships that could be construed as a potential conflict of interest.

## Publisher's Note

All claims expressed in this article are solely those of the authors and do not necessarily represent those of their affiliated organizations, or those of the publisher, the editors and the reviewers. Any product that may be evaluated in this article, or claim that may be made by its manufacturer, is not guaranteed or endorsed by the publisher.
