# Supplementary material for: Benefits of Digital Mental Health Care Interventions for Correctional Workers and Other Public Safety Personnel: A Narrative Review
Source: Front Psychiatry. 2022 Jul 8;13:921527. doi: 10.3389/fpsyt.2022.921527 (PMC9304966; doi:10.3389/fpsyt.2022.921527)
Supplement: Supplementary file 1 [file Data_Sheet_1.docx]

Supplementary Material


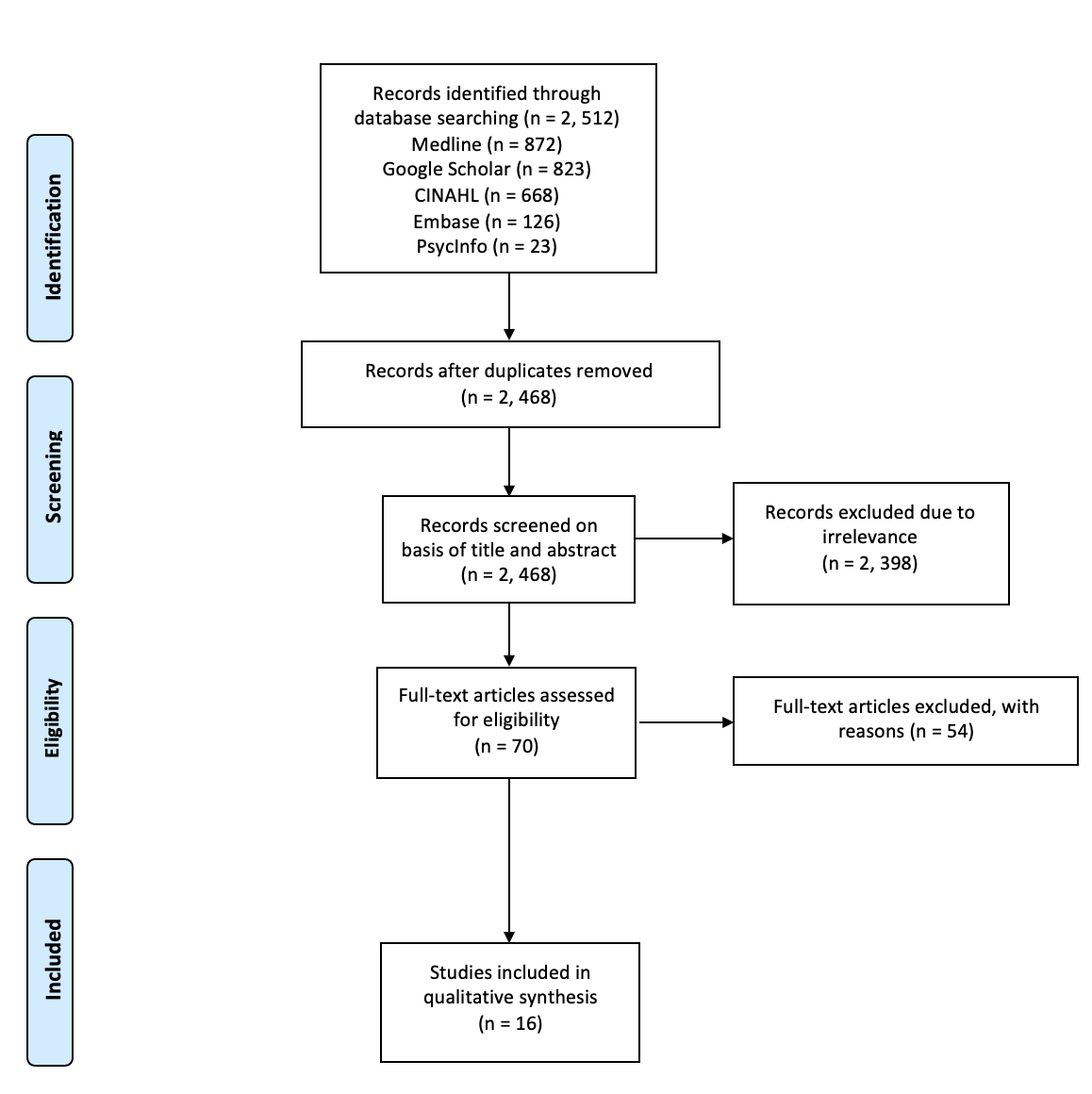


**Figure 1. PRISMA Flow Chart**


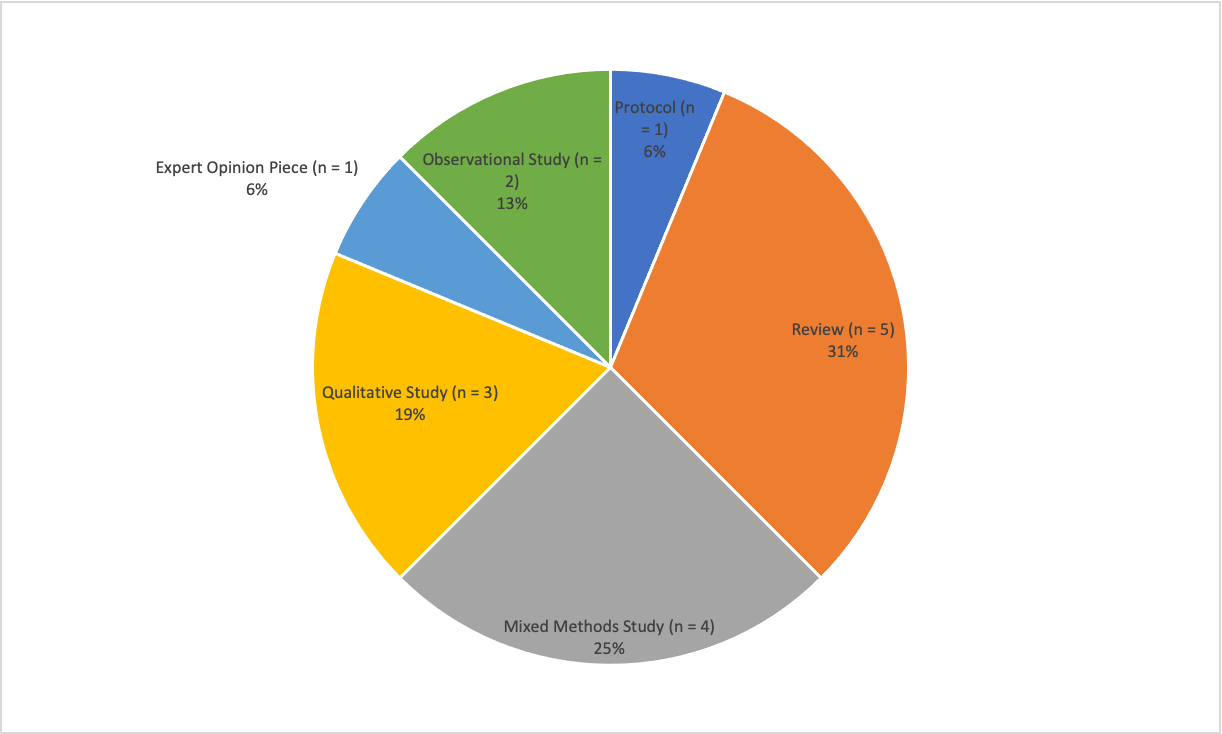


**Figure 2. Type of publication**


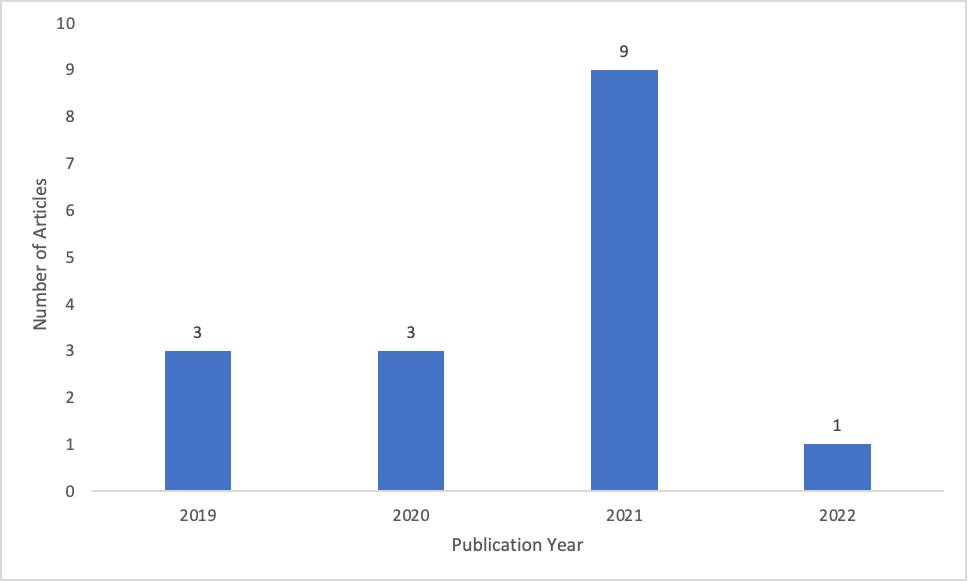


**Figure 3. Articles by publication year**

**Table 1. Subject headings and keyword search terms**

| **Correctional and Public Safety Personnel** | | **Digital Interventions** | | **Mental Health** | |
| --- | --- | --- | --- | --- | --- |
| Subject Headings   - *Prisons* | Keywords   - *Correc* employee* - *Correc* officer* - *Correct* staff* - *Correct* work** - *Detention officer* - *Detention guard* - *Jail officer* - *Jail guard* - *Prison employee* - *Prison staff* - *Prison warden* - *Public safety personnel* | Subject Headings   - *Cell phone* - *Computer* - *Computer communication networks* - *Computer-assisted* - *Computers, handheld* - *Delivery of health care* - *Digital technology* - *Distance counseling* - *Internet* - *Internet access* - *Internet use* - *Internet-based intervention* - *Online social networking* - *Online systems* - *Remote consultation* - *Smartphone* - *Telemedicine* - *Web-based* | Keywords   - *Computer-assisted therapy* - *Digital* - *Digital health* - *Distance counselling* - *E-mental health* - E-mental health - *E-therapy* - *Ehealth* - *I-CBT* - *Internet* - *Internet therapy* - *Internet treatment* - *Mental health services* - *Mhealth* - *Online* - *Online interventions* - *Online therapy* - *Remote consultation* - *Remote therapy* - *Remote training* - *Remote treatment* - *Smartphone* - *Telemedicine* - *Teletherapy* - *Training* - *Web-based* - *Web-based treatment* | Subject Headings   - *Cognitive behavioral therapy* - *Mental health* - *Mental Health Services* - *Psychological trauma* - *Psychotherapy* - *Stress disorders, post-traumatic* - *Therapeutics* - *Training support* | Keywords   - *Cognitive behavioral therapy* - *Counseling* - *Mental health* - *Psychological trauma* - *Psychotherapy* - *Therapy* - *Trauma* |

**Table 2. Study Summary Table**

| **Article** | **Study Design** | **Objective** | **Sample** | **Intervention** | **Rationale/Summary of findings related to digital intervention** |
| --- | --- | --- | --- | --- | --- |
| Alavi et al, 2021 (69) | Protocol | Investigate efficacy of CW-specific online psychotherapy program for CW | Canadian correctional workers (n = 225; all phases) | 12-week e-CBT for CWs on the OPTT platform with therapist guidance. The internet psychotherapy is clinically validated, evidence-based, asynchronous, text and image-based, module driven, and diagnosis-specific. | Determining feasibility and effectiveness of program provides CWs with a resource to address their mental health needs. |
| Almost et al, 2019 (80) | Mixed-methods study | Develop an online e-learning intervention for correctional nurses with aim of implementing it in a provincial context | Canadian correctional RN, RPN and HCM (n = 83; all phases) | Two 30-minute webinars delivered back-to-back for 15 weeks that are also recorded and posted online for easier viewing. The CE training program focuses on mental health and addictions. | Participants were satisfied with convenience of online learning with short webinars. Relevancy, length, and topics of teaching material were viewed positively. Needs expressed were technological comfort or access, speakers versed in correctional nursing, adequate staff, and easier login/attendance. |
| Beahm et al, 2021 (68) | Qualitative study | Examine how PSP experience e-CBT before and after treatment and use insights to improve the program | Canadian PSP (n = 82; n = 9 CW) | 8-week *PSP Online Course,* a transdiagnostic e-CBT course tailored for PSP offered on PSPNET. The program consists of five text and image-based psychoeducational lessons. Nine additional materials are tailored with examples for PSP. Flexible frequencies and durations of therapist support, up to 16 weeks maximum. Access to course is up to one year. | Most found the program beneficial for coping skill development and normalizing mental health experiences. Therapist engagement, content, and activities were valued. Some difficulties completing the course within eight weeks. Some skepticism towards the course based on government sponsorship and research orientation, highlighting importance of trust when developing interventions. |
| Carleton, 2021 (137) | Expert opinion piece | Overview and rationale for series of applied research efforts to improve Canadian PSP mental health care | N/A | Several online programs and interventions discussed. AX1 online survey gathers data on PTSI and risk and resiliency factors. | Data from AX1 survey may help inform solutions to better support PSP health: Examples of digital innovations are, (1) a free online self-report mental disorder screening tool where an individual’s clinical cut-off scores are compared to that of the general population and other PSP; (2) a cost-effective and evidence-based e-CBT program (*PSP Wellbeing Course* on PSPNET) |
| Di Nota et al, 2021 (102) | Systematic review and meta-analysis | Measure effectiveness of proactive mental health programs in PSP exposed to PPTE | Data from 42 studies (7 examining web-based delivery) with a global sample of PSP (n = 3182; n = 117 CW) | Web-based psychoeducation programs including 1. Online Workplace Mental Health Intervention; 2. Web-based stress management program; 3. Road to Mental Readiness; and 4. Stress Management Mobile App | Proactive programs resulted in significant reductions in PTSI. Self-directed or web-based programs had low adherence and completion, although none sampled from CW populations. Implementation of web-based programs may be more cost-effective than multiple in-person group sessions |
| Granek et al, 2019 (29) | Mixed-methods study | Comprehensive evaluation during all stages of the R2MR app development | CAF members recruited for various validation and content assessment studies:  -Content validation study (n = 392)  -Biofeedback and arousal control evaluation (n = 10; n = 28) | A mobile app that complements the current program and provides on-the-go training to assist with stress management, mental health, and treatment seeking. Application consists of customizable life skills, progress tracking, resilience and executive functioning skills, and rapid access to mental health information. Includes multimedia and graphics to enhance engagement | App was more likely to be accepted by CAF members than civilians as a prescribed training tool. Members expressed desire to see their progress relative to others. |
| Hadjistavropoulos et al, 2019 (70) | Observational study | Examine   effectiveness of a tailored transdiagnostic e-CBT program for treating depression, anxiety, and PTSD symptoms in PSP | Canadian PSP (n = 83; n = 9 CW) | 8-week *PSP Online Course,* a transdiagnostic e-CBT course tailored for PSP offered on PSPNET. The program consists of five text and image-based psychoeducational lessons. Nine additional materials are tailored with examples for PSP. Flexible frequencies and durations of therapist support, up to 16 weeks maximum. Access to course is up to one year. | Effective for treating symptoms of depression, anxiety, and PTSD and improved functioning over three domains of life. Large symptoms reductions in measures of depression and anxiety, and moderate in PTSD. Moderately effective for treating anger. Slightly effective in treating social anxiety symptoms. Preference for flexible treatment duration and high working alliance observed. Most accessed additional resources. |
| Jones et al, 2020 (26) | Systematic scoping review | Evaluate scope and quality of peer-reviewed studies on remotely delivered digital mental health interventions for military members, veterans, and PSP. Additionally, determine needs, gaps, and barriers of these interventions | Data from 38 studies with a sample of UK and US-based military members, veterans, and PSP (n = 78; n = 3 PSP; CW = DNR) | Virtual therapies included (1) Motivational interviewing; (2) Behavioural activation treatment; (3) Psychoeducation; (4) Behavioural activation and therapeutic exposure; (5) cognitive behavioural therapy; (6) cognitive processing therapy; (7) prolonged exposure therapy | Virtual delivery can be as effective as in-person. Treatment accessibility is improved due to convenience, low costs and stigma, and remote use at the comfort and safety of the home environment. Personalization and flexibility of therapy should be considered. Additional research examining influences of gender, race, and culture is necessary. |
| McCall et al, 2021A (65) | Qualitative study | Exploring perceptions of e-CBT and how to tailor the program to meet PSP needs | Canadian PSP stakeholders (n = 126; n = 25 in corrections) | Findings were used to guide the development of an e-CBT program tailored for PSP (*PSP Wellbeing Course* offered on PSPNET) | PSP have positive perceptions of e-CBT tailored for them. Privacy was most frequently identified advantage, which addresses stigma. Although accessibility is improved, lack of face-to-face therapist contact, and potential technological problems are disadvantages. PSP prefer programs that address different symptoms, include additional content on other issues and concerns of PSP, has flexible delivery and timelines, and contains therapist contact |
| McCall et al, 2021B (66) | Observational study | Understanding why PSP seek e-CBT | Canadian PSP (n = 259; n = 55 CW) | Data extracted from eligibility screening questionnaires for the *PSP Wellbeing Course* offered on PSPNET | Most learned about PSPNET from employers, colleagues, unions, or professional associations. Reduced stigma may further improve positive perceptions of the program. Common motivations were convenience of program and desire to learn coping skills |
| McCall et al, 2020 (37) | Mixed-methods study | Exploring perceptions of e- CBT when presented with a poster or a poster supplemented with a story of a PSP who benefitted from e- CBT | Canadian PSP (n = 132; n = 18 CW) | Posters and story pertained to the *PSP Wellbeing Course* offered on PSPNET | Participants had positive perceptions of e- CBT. Most would use e- CBT if they needed help with mental health concerns. The program was perceived as moderately credible and acceptable and moderate symptoms improvement and adequate adherence was expected. After psychologists, therapist-guided CBT was the most preferred treatment type. Females found the program more credible than males. Receiving additional information about e- CBT via story did not change level of positive perceptions compared to the poster alone. |
| O’Toole & Brown, 2021 (32) | Systematic review | Evaluation of the quality of mental health apps for resiliency that are specifically targeted for MM and PSP | Apps with a clear mental health component, that cost no more than $5.00 and were specifically developed for MM and PSP (n = 12) | (1) AIMS for Anger Management  (2) Breathe2Relax  (3) Concussion Coach  (4) DoD Safe Helpline  (5) High Res  (6) LifeArmor  (7) Mindfulness Coach  (8) Mood Coach  (9) Moving Forward  (10) PTSD Coach  (11) PTSD Family Coach  (12) Tactical Breather | Most apps were developed by credible government agencies associated with military and Veterans. The content was also population-specific. Only one app was evaluated in an RCT. Majority of the apps require improvements on visuals to explain concepts. Few apps focused on PSP. The apps focused on therapeutic breathing, progressive muscle relaxation, guided imagery, sleep hygiene and education, and mindfulness exercises to improve mental wellbeing and resilience. CBT principals were evident in many of the apps. |
| Smith-MacDonald et al, 2021 (31) | Qualitative study | Explore the experience of digital mental health service providers with digital health services for military, veteran, PSP. | Canadian stakeholders: (1) MM, PSP, and veterans in peer support, health or wellness or mental health service positions; (2) multidisciplinary mental health service providers; (3) organizational leaders and policy and decision-makers from relevant organizations; (4) subject matter experts and researchers (n = 31;  Corrections = DNR) | None. The study examined general views on digital mental health services and technology | All participants reported using digital delivery of mental health services and suggested its potential as a standard mode of trauma therapy. This delivery method was seen as improving accessibility of evidence-based mental health treatments. However, technologies, infrastructure, policies, and procedures, particularly those related to privacy and security, need to be updated. Service providers may also need relevant training and support to deliver quality digital mental health care. A hybrid model combing in-person and digital mental health service may allow for flexible and personalized care delivery. Clients should also receive guidance for greater acceptance of digital mental health services. |
| Stelnicki et al, 2021 (31) | Mixed-methods study | Evaluation of a digital mental health program to enhance self-awareness and healthy relationships | Canadian PSP  (n = 136; n = 0 CW) | BOS is a 16-hr program divided into 8-week group sessions. The program is a combination of CBT and psychoeducation and is facilitated by a mental health clinician. The aim is for participants to become more aware of the physical, mental, and emotional aspects of themselves. Subsequent to the program, 10 follow-up sessions are scheduled with a chance to implement skills in real life and to further review, build, and develop BOS skills | Participants perceived the program positively and there were improvements in PTSD, depression, anxiety, stress, alcohol use symptoms. Greater quality of life, perceived social support, resilience, emotional regulation, and stigma were also observed over time. Participants suggested psychological support for treatment completers, offering different treatment delivery methods, introducing coping skills early in the program, biweekly instead of monthly follow-up sessions, participation early in PSP careers, and flexible delivery to account for shift work. |
| Vermetten et al, 2020 (33) | Review | Review applications for MM and Veterans that increase mental health literacy, overcome barriers to care, and enhance well-being and performance | Smartphone mental health apps (n = 4) | (1) R2MR: training tool based on CBT  (2) Unit Victor: chat support for Veterans  (3) UrMMind (pre-deployment): reinforce healthy behaviours and teach coping skills  (4) iFeel: passive collection and analyzation of cellphone data to detect early signs of depression | Mobile apps have an increasingly innovative role in mental health care. Correct design and integration can render many benefits. However, security and privacy must be adequately addressed |
| Voth et al, 2022 (34) | Scoping review | Scoping review of current evidence base to evaluate quality, efficacy, and effectiveness of mobile apps for MMs, PSP, and Veterans on resilience-building and self-regulatory strategies | Mobile apps for MM and PSP (n = 22) | The mobile apps were available on the Apple App Store and Google Play, were free to download, and were meant for use by MM or PSP. | Most of the mHealth apps reviewed provided adequate resilience strategies and skills for MMs, PSP, and Veterans. Frequent resilience strategies included mindfulness, psychoeducation, and positive coping or thinking skills. Majority of the apps were developed by credible, military-focused government agencies. Only two apps assessed were developed outside of Canada. Half of the apps were evidence-based, and more than half used evidence-based strategies. Most of the apps were text-heavy which could be disadvantageous for those with different learning styles. Motivation, app use literacy, access to social support, use of app with an HCP or independently can also impact resilience. These easily accessible apps can encourage help-seeking behaviours in users, particularly when stigma or uncertainty prevent care. |

Note: BOS = Before Operational Stress; CAF = Canadian Armed Forces CE = continuing education; CW = correctional worker; DNR = did not report; e-CBT = electronic cognitive behavioural therapy; OPTT = Online Psychotherapy Tool; R2MR = Road to Mental Readiness program; RN = registered nurse; RPN = registered nurse practitioner; HCM: correctional healthcare manager; HCP = healthcare professional; MM = military members; PPTE = potentially psychologically traumatic exposure; PSP = public safety personnel; PTSD = post-traumatic stress disorder; PTSI = post-traumatic stress injury
